# Trends and causes of maternal mortality in Taiyuan, China, 2016–2024: A population-based observational study

**DOI:** 10.1371/journal.pone.0362983

**Published:** 2026-11-13

**Authors:** Fang Wang, Hailan Yang

**Affiliations:** Department of Obstetrics, The First Hospital of Shanxi Medical University (First Clinical Medicine College), Taiyuan, Shanxi, China; Federal Medical Centre Birnin Kudu, NIGERIA

## Abstract

**Background:**

Although China has made major advances in maternal health, city-level epidemiological evidence on maternal mortality remains scarce. Using maternal surveillance data from Taiyuan (2016–2024), we assessed trends in the maternal mortality ratio (MMR), examined causes of death, and identified associated factors to inform region-specific strategies for reducing MMR.

**Methods:**

We extracted and validated data on maternal deaths and live births from the *Taiyuan Municipal Maternal and Child Health Annual Reports*. MMR was calculated per 100,000 live births. Poisson regression was used to examine temporal trends, and chi-square or Fisher’s exact tests were used for between-group comparisons. Analyses were stratified by geographic region, cause of death, and place of death; the study period was divided into three phases (2016–2018, 2019–2021, 2022–2024) for subgroup analysis.

**Results:**

Between 2016 and 2024, Taiyuan recorded 424,392 live births and 33 maternal deaths, yielding an overall MMR of 7.76 per 100,000 live births (95% CI 5.35 to 10.92). Urban MMR declined significantly (P < 0.0001), whereas rural MMR showed no significant temporal change (P = 0.313) and remained higher than urban levels (9.57 vs 7.58 per 100,000). Indirect obstetric causes accounted for 66.7% of maternal deaths, compared with 33.3% for direct causes, with no significant temporal trends in either category. Women aged 30–39 years accounted for 63.6% of maternal deaths, and 83.9% of deaths occurred in tertiary hospitals.

**Conclusion:**

Taiyuan has maintained a low MMR through sustained investment in maternal healthcare; however, substantial urban-rural disparities remain. Indirect obstetric causes predominated among maternal deaths, suggesting a transition in the spectrum of maternal causes of death. Continued efforts to strengthen rural maternal health services and promote equitable, life-course-oriented maternal care are essential to further reduce preventable maternal deaths.

## Introduction

MMR has been widely used as a measure to assess the quality of care and advances in maternal care [1]. Reducing maternal mortality has emerged as a critical priority for global public health, reflected in its designation as a principal objective within the Millennium Development Goals (MDGs) [2] and the Sustainable Development Goals (SDGs) [3]. China has achieved the MDG target for maternal health and met the SDG 3.1 MMR threshold.The Healthy China 2030 Plan Outline sets a national target of reducing the MMR to 12 deaths per 100,000 live births by 2030 [4]. Nevertheless, according to 2024 monitoring data, China's MMR remains at 14.3 per 100,000 live births, indicating that sustained, targeted, and system-level efforts are still required to achieve this goal. Given China’s large population, the absolute number of maternal deaths remains substantial, underscoring considerable scope for preventing avoidable maternal deaths [5,6]. Marked regional disparities in the MMR persist across China: MMR is higher in rural versus urban settings and in remote compared with coastal regions [7,8]. The remarkable achievements in Maternal and Child Health (MCH) in China may be attributed to the rapid socioeconomic development since China's reform and opening-up, the significant improvement in MCH medical services, and effective government policies dedicated to MCH [9], key initiatives include: featuring in the subsidies and health education, maternal hospital delivery insurance reimbursements, the rural maternal hospital delivery subsidy program, the provision of five free prenatal check-ups and the green channel service for obstetric emergencies, among other support measures [10].

In October 2015, China implemented the Universal Two-Child Policy, and in May 2021, China officially introduced the Three-Child Policy. These policies may have contributed to an increase in the proportion of advanced maternal age (AMA) pregnancies. Studies have shown that as maternal age increases, the risk of gestational hypertension, gestational diabetes, placenta previa, and severe postpartum hemorrhage, among other complications, also increases [11,12]. China launched the Five Strategies for Maternal and Newborn Safety (FSMNS) in 2017 as an integral part of its ambitious national public health program within the framework of Healthy China 2030. The core constituents of FSMNS include risk screening and assessment, risk-graded management and referral, case-specific management of high-risk pregnancies, strict reporting mechanisms, and a robust system of accountability [13]. The FSMNS represents a significant institutional innovation in China's maternal and child health work. This approach classifies pregnant women into five color-coded levels—green (low risk), yellow (moderate risk), orange (high risk), red (severe risk), and purple (infectious disease risk)—based on health status and pregnancy-related risks, enabling dynamic monitoring and tiered management. Risk assessment and color coding commence when a pregnant woman establishes her Maternal Health Handbook at primary healthcare institutions. The policy mandates referral to tertiary hospitals for pregnant women at high risk (orange or red). This framework facilitates timely intervention, promotes precise allocation of medical resources, and supports an efficient referral and treatment network for high-risk pregnancies.

As the political, economic, cultural, and international exchange hub of Shanxi Province—and its most populous city—Taiyuan occupies a central position in the regional health-care delivery system. Its distinctive urban-rural population structure provides a valuable setting for examining regional maternal health dynamics. However, long-term, systematic surveillance data on the epidemiology of maternal mortality in Taiyuan are lacking. To address this gap, we analyzed maternal mortality trends in Taiyuan from 2016 to 2024, assessed urban—rural disparities, examined shifts in the leading causes of maternal death, and evaluated the implementation and performance of local maternal health policies. This study seeks to identify key patterns, clarify ongoing challenges, and produce evidence-based recommendations to enhance obstetric emergency response, improve prenatal, intrapartum, and postpartum care, and strengthen maternal and neonatal safety mechanisms. These findings may offer practical insights that support sustained progress in maternal health in Taiyuan and other rapidly urbanizing regions across China.

## Materials and methods

### Data source

Data were extracted from the *Taiyuan Maternal and Child Health Annual Statistical Report*. Routine, population-based maternal mortality surveillance was implemented in Taiyuan following the Shanxi Provincial Maternal Mortality Monitoring Program and Taiyuan Municipal Implementation Rules for Maternal Mortality Monitoring. Full-time staff at the Taiyuan Maternal Health and Family Planning Service Center performed surveillance data collection, quality control, statistical analysis, and municipal-level maternal mortality reviews; aggregated findings from this surveillance were compiled into the annual report. We analysed live births and maternal deaths reported by all medical institutions across Taiyuan.

### Definitions

Maternal death is defined as the death of a woman while pregnant or within 42 days of termination of pregnancy, irrespective of the duration and the site of the pregnancy, from any cause related to or aggravated by pregnancy or its management, but not from accidental or incidental causes [14].

We used the standard maternal mortality ratio (MMR = maternal deaths/100,000 live births) to record maternal deaths over time.

Direct obstetric death is defined as maternal death caused by obstetric complications in pregnancy (pregnancy, childbirth and puerperium) or medical operation intervention, negligence and improper handling, such as amniotic fluid embolism, ectopic pregnancy, pregnancy-induced hypertension, etc.

Indirect obstetric death is defined as maternal death caused by previous diseases or new diseases during pregnancy. Although these diseases are not caused by direct obstetric reasons, they are aggravated by the physiological effects of pregnancy, such as congenital heart disease, pulmonary tuberculosis, liver cirrhosis, etc.

### Statistical analysis

After thorough data cleaning and coding protocols, all statistical analyses were conducted using R version 4.5.0 for Windows. Poisson regression was used to quantify temporal trends in MMR from 2016 to 2024, with log-transformed annual live births as an offset. The model was specified as log(*μt*)=log (LB*μt* + *β0* + *β1* × year_t_, where *μ*_*t*_ denotes the expected number of maternal deaths in year t. The annual percent change (APC) was derived as (exp(*β*_*1*_) − 1) ×100%, with its 95% confidence interval calculated from the standard error of *β*_*1*_.Overdispersion was assessed by dividing the Pearson chi-square statistic by its degrees of freedom; a value > 1.5 indicated overdispersion, in which case negative binomial regression was used as an alternative. All reported *P* values were two-sided, with P < 0.05 considered statistically significant. All MMR point estimates are reported with 95% CIs calculated using the exact Poisson method. The nine-year investigation period (2016–2024) was stratified into three distinct chronological phases (2016–2018, 2019–2021, and 2022–2024) to facilitate comparative analysis. Inter-period MMR variations were assessed using Chi-square tests or Fisher's exact tests.

### Quality control

Maternal mortality monitoring has become a routine practice, with a three-tiered network operating at the county, township, and village levels. This network adheres strictly to the Taiyuan Maternal Mortality Monitoring Program, ensuring that reporting personnel are trained and assessed for their duties. Maternal death report cards, completed by full-time staff at health-care institutions at or above the county

level, must be accurate and complete, with no omissions, vacancies, or logical errors. A detailed case summary is required for each maternal death. The local maternal mortality review committee conducts reviews every six months; each case undergoes standardized review, and an individual report is prepared to identify the most likely cause of death, preventable factors, and recommended interventions. Causes of death were coded according to the ICD-10.Ethical approval was approved by the Academic Committee of The First Hospital of Shanxi Medical University and the academic committee have granted permission to conduct the study.The ethical approval number is KYLL-2026–439. All data used in this study were de-identified aggregated routine surveillance data, with no access to individual identifiable personal information.

## Results

### Trends of the MMR in Taiyuan from 2016 to 2024

From 2016 to 2024, the MMR in Taiyuan, Shanxi Province, and at the national level exhibited distinct temporal patterns (Fig 1). The national MMR demonstrated a consistent downward trend throughout the study period. In contrast, Shanxi Province experienced year-to-year fluctuations in its MMR, which reached its lowest level in 2022 at 3.22 per 100,000 live births. Similarly, the MMR in Taiyuan varied over time, and no maternal deaths were recorded in 2022 and 2024. Annual live births in Taiyuan peaked in 2017, totalling 59,407 (Table 1).

**Table 1:** The MMR in urban and rural areas of Taiyuan during 2016–2024.

Year	Rural Area				Urban Area				Total			
	Live births	Deaths	MMR	95% CI	Live births	Deaths	MMR	95% CI	Live births	Deaths	MMR	95% CI
2016	5,236	1	19.09	(0.48, 106.36)	42,750	8	18.71	(8.08, 36.86)	47,986	9	18.76	(8.58, 35.60)
2017	5,721	0	0.00	(0.00, 64.45)	53,686	7	13.04	(5.24, 26.86)	59,407	7	11.78	(4.74, 24.28)
2018	5,151	1	19.42	(0.49, 108.19)	46,865	7	14.94	(6.01, 30.78)	52,016	8	15.38	(6.64, 30.30)
2019	7,368	1	13.57	(0.34, 75.61)	49,376	2	4.05	(0.49, 14.63)	56,744	3	5.29	(1.09, 15.45)
2020	4,228	1	23.65	(0.60, 131.78)	40,570	2	4.93	(0.60, 17.81)	44,798	3	6.70	(1.38, 19.57)
2021	3,708	0	0.00	(0.00, 99.47)	36,364	2	5.50	(0.67, 19.87)	40,072	2	4.99	(0.60, 18.03)
2022	3,591	0	0.00	(0.00, 102.75)	37,750	0	0.00	(0.00, 9.77)	41,341	0	0.00	(0.00, 8.92)
2023	3,207	0	0.00	(0.00, 115.04)	33,922	1	2.95	(0.07, 16.43)	37,129	1	2.69	(0.07, 15.01)
2024	3,607	0	0.00	(0.00, 102.29)	41,292	0	0.00	(0.00, 8.94)	44,899	0	0.00	(0.00, 8.22)
**Total**	**41,817**	**4**	**9.57**	**(2.61, 24.49)**	**382,575**	**29**	**7.58**	**(5.08, 10.89)**	**424,392**	**33**	**7.78**	**(5.35, 10.92)**

Notes: MMR: Maternal Mortality Ratio (per 100,000 live births). 95% CI: confidence intervals calculated using the Poisson exact method.

**Fig 1:**
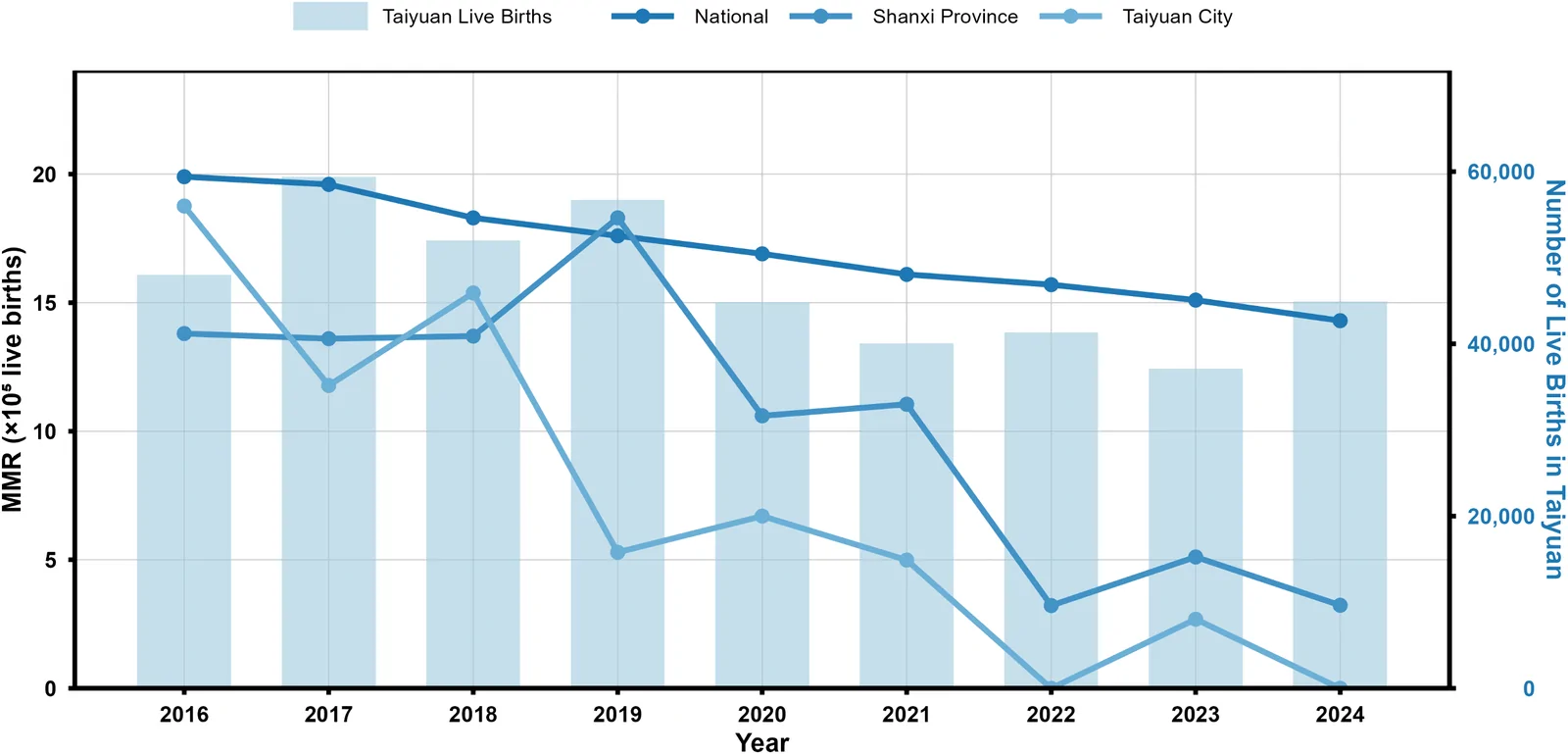
MMR trends and patient count (2016–2024) National MMR declined consistently, Shanxi MMR fluctuated with the 2022 nadir (3.22/10^5^), and Taiyuan MMR varied with the 2019 minimum (5.29/10^5^) and zero deaths in 2022 and 2024.

Throughout the study period, Taiyuan recorded 424,392 live births and 33 maternal deaths, resulting in an overall MMR of 7.76 per 100,000 live births(95% CI: 5.35 to 10.92). The highest annual MMR occurred in 2016 (18.76 per 100,000 live births), while the lowest rates were observed in 2022 and 2024 (0.00 per 100,000 live births) (Fig 2). Taiyuan consistently maintained MMR levels below the national average, except for 2016 and 2018. Compared with the Shanxi provincial, Taiyuan had higher MMR in 2016, 2018, and 2023, with lower values across all other study years (Table 2).

**Table 2:** The MMr due to direct and indirect obstetric causes in Taiyuan during 2016–2024.

Year	Direct Obstetric Causes			Indirect Obstetric Causes		
	Deaths (n)	MMR	95% CI	Deaths (n)	MMR	95% CI
2016	5	10.42	(3.38, 24.32)	4	8.34	(2.27, 21.34)
2017	1	1.68	(0.04, 9.38)	6	10.10	(3.71, 21.98)
2018	2	3.84	(0.47, 13.89)	6	11.53	(4.23, 25.11)
2019	2	3.52	(0.43, 12.73)	1	1.76	(0.04, 9.82)
2020	1	2.23	(0.06, 12.44)	2	4.46	(0.54, 16.13)
2021	0	0.00	(0.00, 9.21)	2	4.99	(0.60, 18.03)
2022	0	0.00	(0.00, 8.92)	0	0.00	(0.00, 8.92)
2023	0	0.00	(0.00, 9.94)	1	2.69	(0.07, 15.01)
2024	0	0.00	(0.00, 8.22)	0	0.00	(0.00, 8.22)
**Total**	**11**	**2.59**	**(1.29, 4.64)**	**22**	**5.18**	**(3.25, 7.85)**

Notes

Trends were analyzed by Poisson regression models with log(live births) as an offset. Direct obstetric causes showed a significant downward trend (APC = −40.00%, 95% CI: −58.58% to −13.09%, P = 0.0069), and indirect obstetric causes also showed a significant downward trend (APC = −25.43%, 95% CI: −39.09% to −8.69%, P = 0.0045).

**Fig 2:**
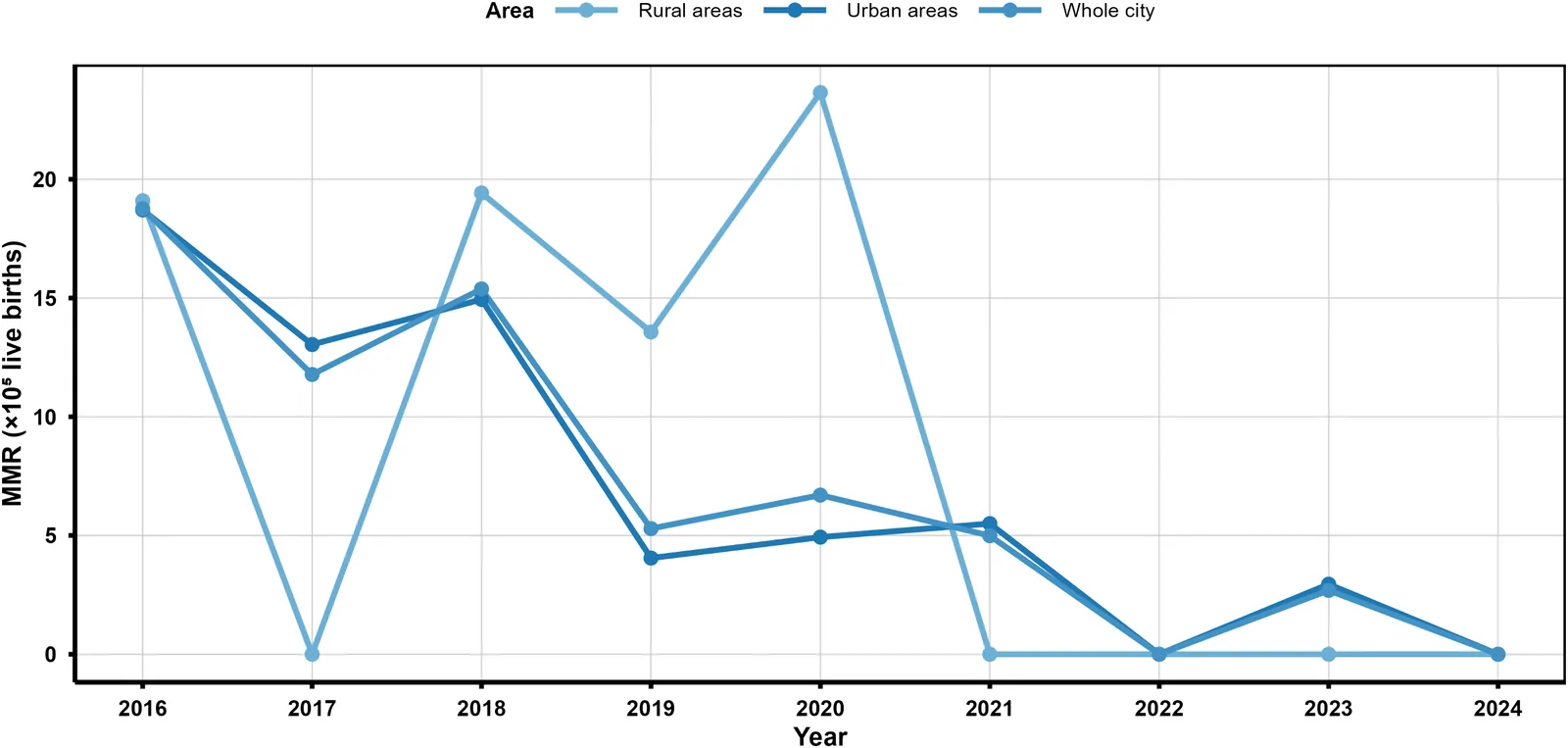
MMR trends in Taiyuan (2016–2024). Trends were analyzed by Poisson regression models with log (live births) as an offset. The overall MMR showed a significant downward trend (APC = −29.83%, 95% CI: −41.22% to −16.23%, P = 0.0001). Urban areas also showed a significant decline (APC = −30.89%, 95% CI: −42.98% to −16.23%, P = 0.0002), while rural areas showed no significant trend (APC = −22.70%, 95% CI: −51.05% to 22.06%, P = 0.2693).

The MMR varied between urban and rural areas. Rural regions recorded 41,817 live births and 4 maternal deaths, resulting in an MMR of 9.57 per 100,000 live births. In contrast, urban areas reported 382,575 live births with 29 maternal deaths, yielding an MMR of 7.58 per 100,000 live births. Poisson regression analysis revealed a significant downward trend in overall MMR over the study period (APC = −29.83%, 95% CI: −41.22% to −16.23%, P = 0.0001). Urban areas showed a significant decline (APC = −30.89%, 95% CI: −42.98% to −16.23%, P = 0.0002), while rural areas showed no significant trend (APC = −22.70%, 95% CI: −51.05% to 22.06%, P = 0.2693). Chi-square analyses indicated no statistically significant differences in MMR between urban and rural areas, whereas subgroup analysis revealed a statistically significant urban–rural difference in MMR during 2016–2018 (P < 0.001) (Fig 3).

**Fig 3:**
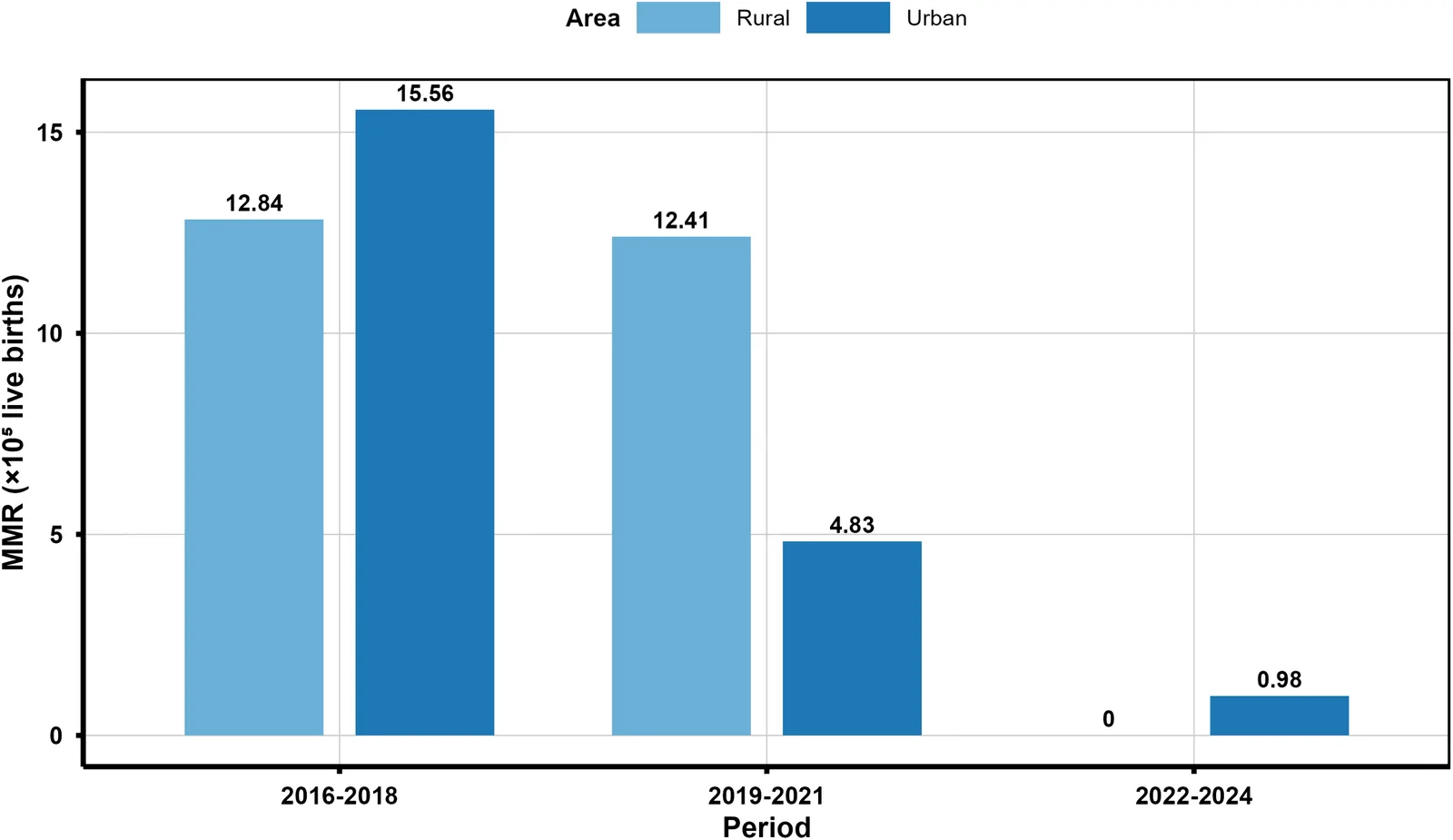
Differences in the MMR between rural and urban areas in different periods. Differences between rural and urban areas were analyzed by Chi-square test. Significant differences were observed in 2016–2018 (P = 0.0001), but not in 2019–2021 (P = 0.289) or 2022–2024 (P = 1.000).

### Trends of the MMR due to direct and indirect obstetric causes in Taiyuan from 2016 to 2024

During the period, a total of 33 maternal deaths were recorded: 11 (33.3%) were attributed to direct obstetric causes and 22 (66.7%) to indirect causes. The MMR for direct causes peaked in 2016 at 10.42 per 100,000 live births, while the peak for indirect causes occurred in 2018 (11.53 per 100,000). Years with zero maternal deaths from direct causes were 2020, 2021, 2022, and 2024. Years with zero maternal deaths from indirect causes were 2022, 2023, and 2024 (Fig 4).

**Fig 4:**
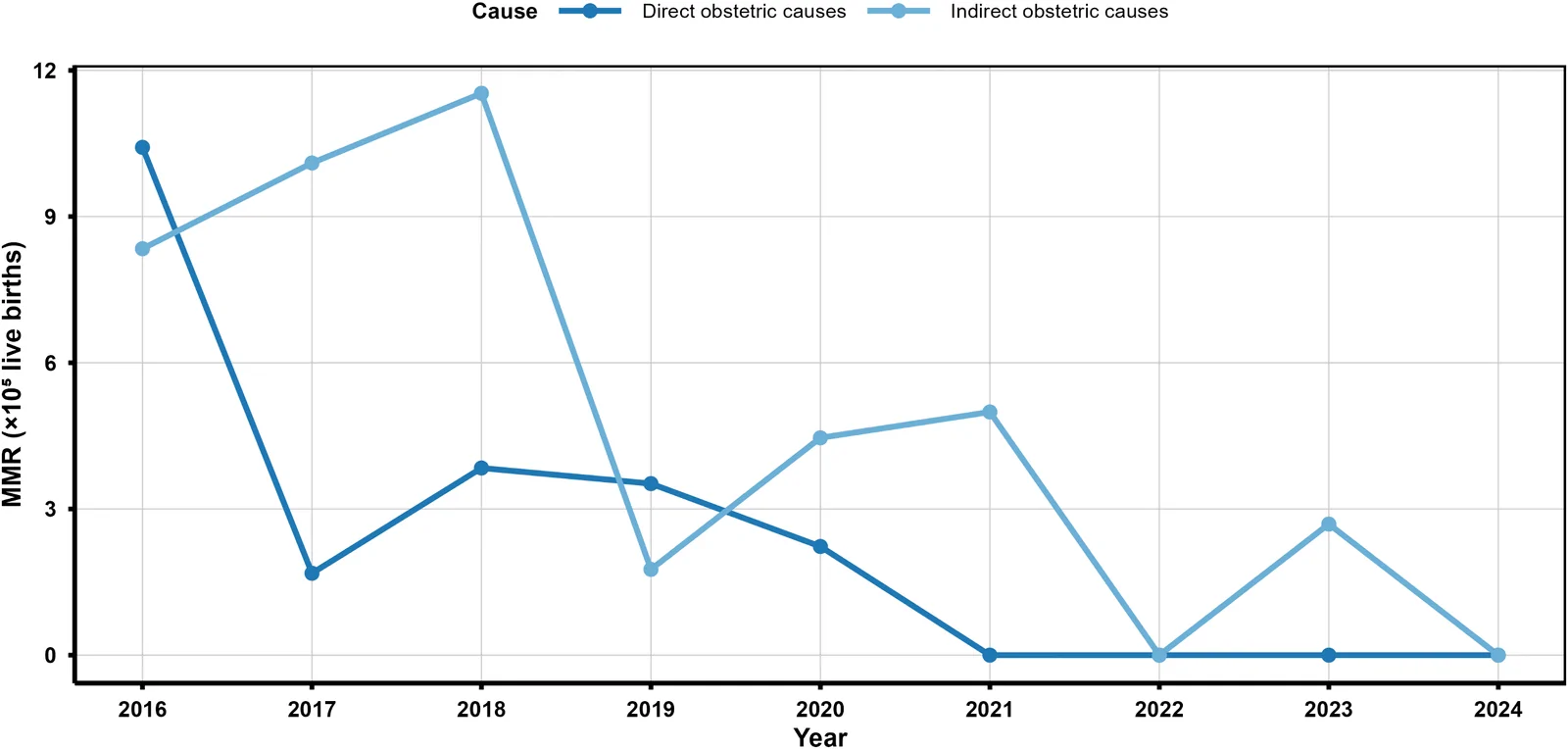
Trends of the MMR due to direct and indirect obstetric causes in Taiyuan (2016–2024). Direct obstetric causes showed a significant downward trend (APC = −40.00%, 95% CI: −58.58% to −13.09%, P = 0.0069), and indirect obstetric causes also showed a significant downward trend (APC = −25.43%, 95% CI: −39.09% to −8.69%, P = 0.0045). Chi-square tests comparing direct vs. indirect causes within each period showed: 2016–2018: P = 0.043, 2019–2021: P = 0.617, 2022–2024: P = 1.000.

Among the direct obstetric causes, hypertensive disorders of pregnancy and amniotic fluid embolism were the most prevalent, each accounting for 3 cases (9.1% of all deaths). Among indirect causes, other medical conditions were the leading contributors (5, 15.2%), followed by cardiovascular disease, cerebral hemorrhage, and thromboembolism, each accounting for 4 cases (12.1%) (Table 4). Indirect obstetric causes accounted for the majority of maternal deaths throughout the study period. Poisson regression analysis revealed a significant downward trend for direct obstetric causes (APC = −40.00%, 95% CI: −58.58% to −13.09%, P = 0.0069) and for indirect obstetric causes (APC = −25.43%, 95% CI: −39.09% to −8.69%, P = 0.0045). Chi-square tests comparing direct and indirect obstetric causes within each time period showed a statistically significant difference only in 2016–2018 (P = 0.043) (Fig 5).

**Fig 5:**
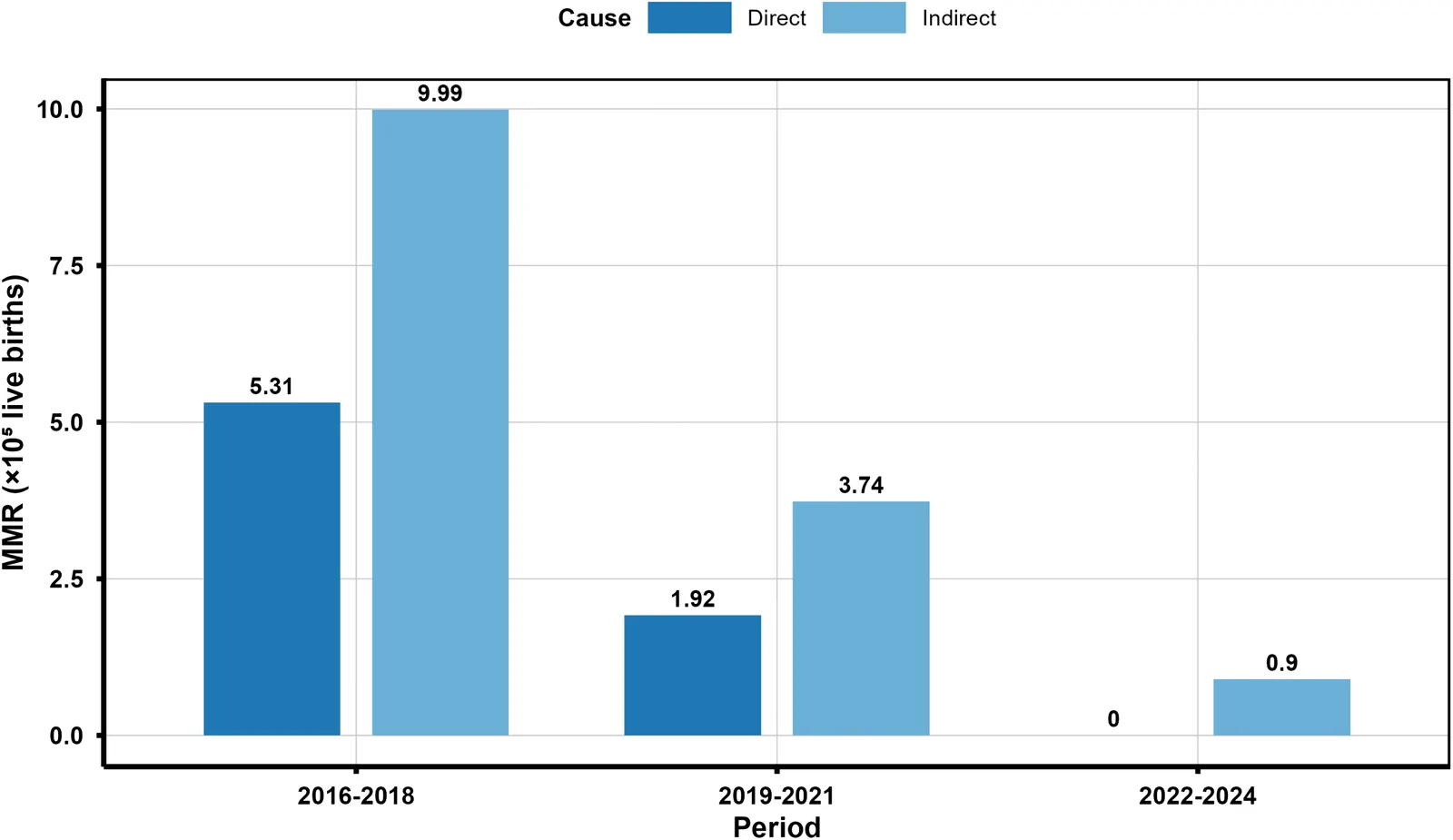
Differences in the MMR due to direct and indirect obstetric causes in different periods. Grouped bar chart showing average MMR for direct and indirect causes across three time periods. Chi-square tests comparing direct vs. indirect causes within each period showed: 2016-2018: P = 0.043, 2019-2021: P = 0.617, 2022-2024: P = 1.000.

### Trends in the age-specific constituent ratio of maternal deaths in Taiyuan from 2016 to 2024

The 30–34 years age group had the highest number of deaths (11 cases, 33.3%), closely followed by the 35–39 years group (10 cases, 30.3%). Collectively, women aged 30–39 years accounted for 21 of the 33 maternal deaths, representing 63.6% of all fatalities during the period (Fig 6).

**Fig 6:**
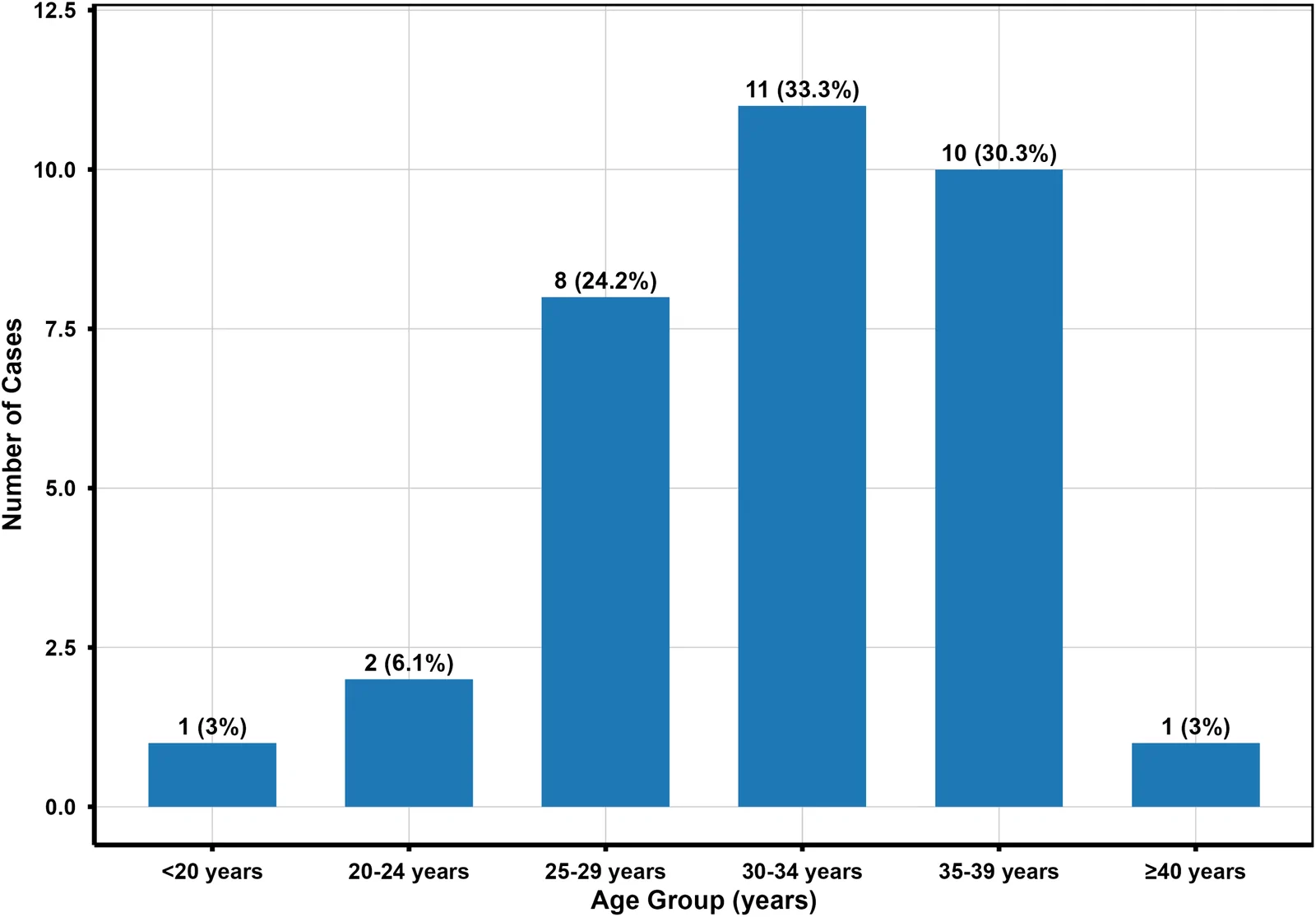
Age distribution of maternal deaths in Taiyuan(2016-2024). The figure presents the number and percentage of maternal deaths by age group during the study period.

### Trends of the constituent ratio in sites of maternal deaths in Taiyuan from 2016 to 2024

Maternal deaths occurred predominantly in provincial/municipal hospitals (26, 83.9% of location-specified deaths), followed by deaths at home (5, 16.1%), during transport (1, 3.2%), and in county hospitals (1, 3.2%); No maternal deaths were reported in township hospitals (Table 3). No maternal deaths were recorded in 2022 or 2024, and only one death (occurring at home) was reported in 2023. The proportional distribution of deaths by location varied across years. Deaths in provincial/municipal hospitals accounted for the majority in most years, comprising 100.0% in 2017, 2019, and 2021, but 0.0% in 2022 and 2024 (with the single death in 2023 occurring at home) (Fig 7).

**Table 3:** The constituent ratio on sites of maternal deaths in Taiyuan from 2016 to 2024 (%).

Year	Home		In transport		County hospital		Provincial/municipal hospital	
	n	%	n	%	n	%	n	%
2016	1	11.1	1	11.1	0	0.0	7	77.8
2017	0	0.0	0	0.0	0	0.0	7	100.0
2018	1	12.5	0	0.0	1	12.5	6	75.0
2019	0	0.0	0	0.0	0	0.0	3	100.0
2020	2	66.7	0	0.0	0	0.0	1	33.3
2021	0	0.0	0	0.0	0	0.0	2	100.0
2022	0	0.0	0	0.0	0	0.0	0	0.0
2023	1	100.0	0	0.0	0	0.0	0	0.0
2024	0	0.0	0	0.0	0	0.0	0	0.0

Notes

n. Number of maternal deaths.

%: Constituent ratio (percentage).

**Fig 7:**
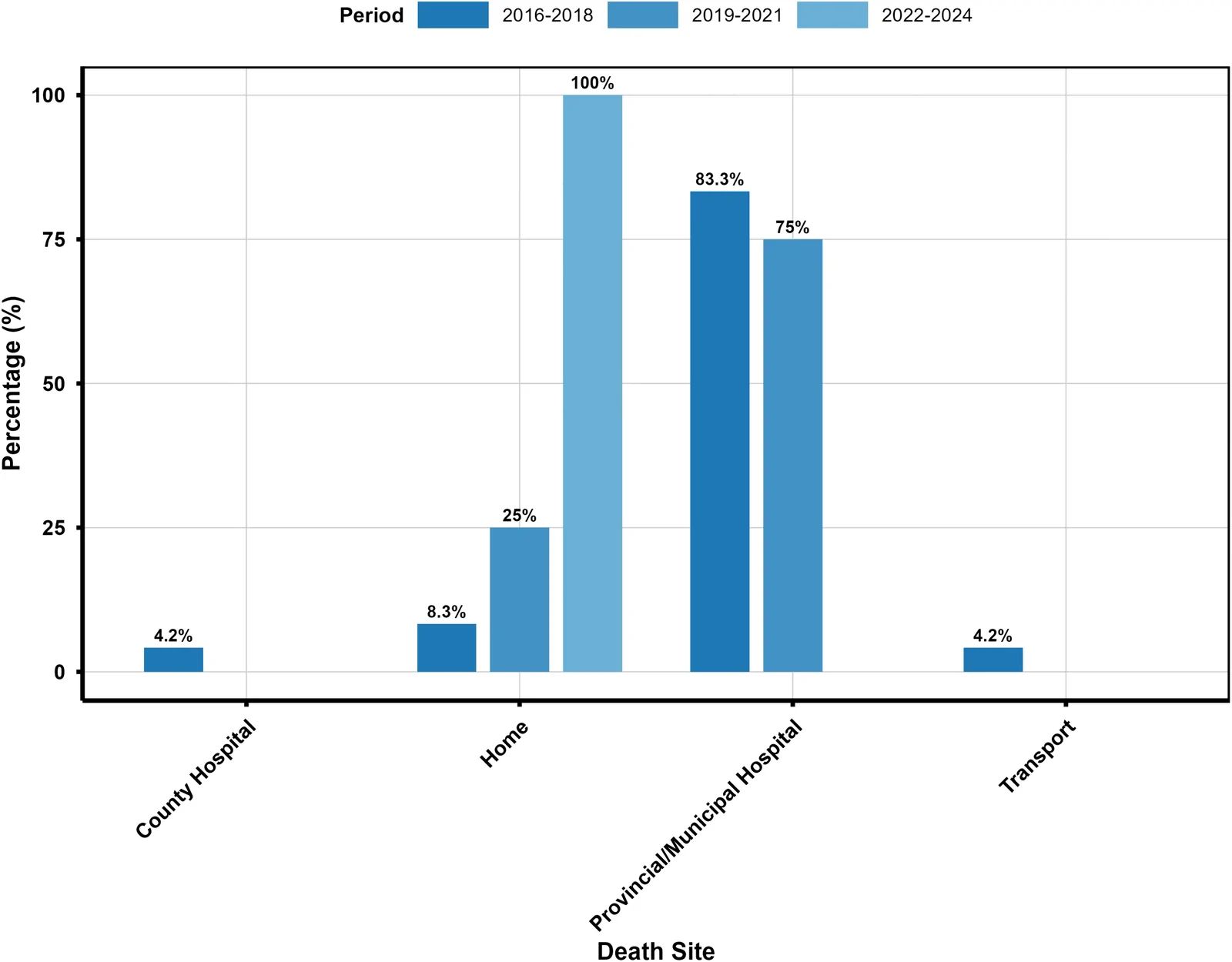
Differences in the constituent ratio of maternal death sites in different periods. No statistical trend tests were performed for constituent ratios as this figure presents proportional distributions.

### Maternal death causes in Taiyuan from 2016 to 2024

During this period, the causes of maternal deaths were categorized into 16 distinct groups (Fig 8). The six leading causes are illustrated in Fig 9: other medical diseases (5, 15.2%), cardiovascular disease (4, 12.1%), cerebral hemorrhage (4, 12.1%), thromboembolism (4, 12.1%), amniotic fluid embolism (3, 9.1%), and hypertensive disorders of pregnancy (3, 9.1%) (Table 4). Together, these causes accounted for 23 of the 33 maternal deaths (69.7%). As indicated in Table 5, the Cochran-Armitage trend test revealed no statistically significant temporal trends for any of these leading causes (P > 0.05).

**Table 4:** Characteristics of maternal deaths in Taiyuan during 2016–2024.

Category	Subcategory	Total Cases (n = 33)	Composition Ratio (%)	2016	2017	2018	2019	2020	2021	2022	2023	2024
Direct Causes	Hypertensive Disorders of pregnancy	3	9.1	1	0	1	0	1	0	0	0	0
	Amniotic Fluid Embolism	3	9.1	1	0	0	2	0	0	0	0	0
	Puerperal Heatstroke	1	3.0	0	0	1	0	0	0	0	0	0
	Intrauterine Fetal death with DIC	1	3.0	1	0	0	0	0	0	0	0	0
	Placenta Previa	1	3.0	1	0	0	0	0	0	0	0	0
	Postpartum Hemorrhage	1	3.0	1	0	0	0	0	0	0	0	0
	Ectopic Pregnancy	1	3.0	0	1	0	0	0	0	0	0	0
	**Direct Causes Subtotal**	11	33.3	5	1	2	2	1	0	0	0	0
Indirect Causes	Other Diseases	5	15.2	1	2	1	0	0	0	0	1	0
	Cardiovascular Disease	4	12.1	0	1	2	0	0	1	0	0	0
	Cerebral Hemorrhage	4	12.1	0	1	0	1	1	1	0	0	0
	Thromboembolism	4	12.1	2	1	1	0	0	0	0	0	0
	Pneumothorax	1	3.0	0	0	0	0	1	0	0	0	0
	Malignant Tumor	1	3.0	1	0	0	0	0	0	0	0	0
	Anesthesia-related Anaphylaxis	1	3.0	0	0	1	0	0	0	0	0	0
	Acute Fatty Liver of pregnancy	1	3.0	0	1	0	0	0	0	0	0	0
	Pneumonia	1	3.0	0	0	1	0	0	0	0	0	0
	**Indirect Causes Subtotal**	22	66.7	4	6	6	1	2	2	0	1	0
**Total**		**33**	**100.0**	**9**	**7**	**8**	**3**	**3**	**2**	**0**	**1**	**0**

**Table 5:** Trends of the MMR in the top 6 causes of maternal deaths.

Year	Other Diseases		Cardiovascular Disease		Cerebral Hemorrhage		Thromboembolism		Amniotic Fluid Embolism			Hypertensive Disorders of pregnancy
	n	MMR (95% CI)	n	MMR (95% CI)	n	MMR (95% CI)	n	MMR (95% CI)	n	MMR (95% CI)	n	MMR (95% CI)
2016	1	2.08 (0.05, 11.61)	0	0.00 (0.00, 7.69)	0	0.00 (0.00, 7.69)	2	4.17 (0.51, 15.06)	1	2.08 (0.05, 11.61)	1	2.08 (0.05, 11.61)
2017	2	3.37 (0.41, 12.16)	1	1.68 (0.04, 9.38)	1	1.68 (0.04, 9.38)	1	1.68 (0.04, 9.38)	0	0.00 (0.00, 6.21)	0	0.00 (0.00, 6.21)
2018	1	1.92 (0.05, 10.71)	2	3.84 (0.47, 13.89)	0	0.00 (0.00, 7.09)	1	1.92 (0.05, 10.71)	0	0.00 (0.00, 7.09)	1	1.92 (0.05, 10.71)
2019	0	0.00 (0.00, 6.50)	0	0.00 (0.00, 6.50)	1	1.76 (0.04, 9.82)	0	0.00 (0.00, 6.50)	2	3.52 (0.43, 12.73)	0	0.00 (0.00, 6.50)
2020	0	0.00 (0.00, 8.23)	0	0.00 (0.00, 8.23)	1	2.23 (0.06, 12.44)	0	0.00 (0.00, 8.23)	0	0.00 (0.00, 8.23)	1	2.23 (0.06, 12.44)
2021	0	0.00 (0.00, 9.21)	1	2.50 (0.06, 13.90)	1	2.50 (0.06, 13.90)	0	0.00 (0.00, 9.21)	0	0.00 (0.00, 9.21)	0	0.00 (0.00, 9.21)
2022	0	0.00 (0.00, 8.92)	0	0.00 (0.00, 8.92)	0	0.00 (0.00, 8.92)	0	0.00 (0.00, 8.92)	0	0.00 (0.00, 8.92)	0	0.00 (0.00, 8.92)
2023	1	2.69 (0.07, 15.01)	0	0.00 (0.00, 9.94)	0	0.00 (0.00, 9.94)	0	0.00 (0.00, 9.94)	0	0.00 (0.00, 9.94)	0	0.00 (0.00, 9.94)
2024	0	0.00 (0.00, 8.22)	0	0.00 (0.00, 8.22)	0	0.00 (0.00, 8.22)	0	0.00 (0.00, 8.22)	0	0.00 (0.00, 8.22)	0	0.00 (0.00, 8.22)

Notes:

Trends were analyzed by Poisson regression models with log (live births) as an offset and showed no statistically significant trends for any of these causes over time (all P > 0.05).

**Fig 8:**
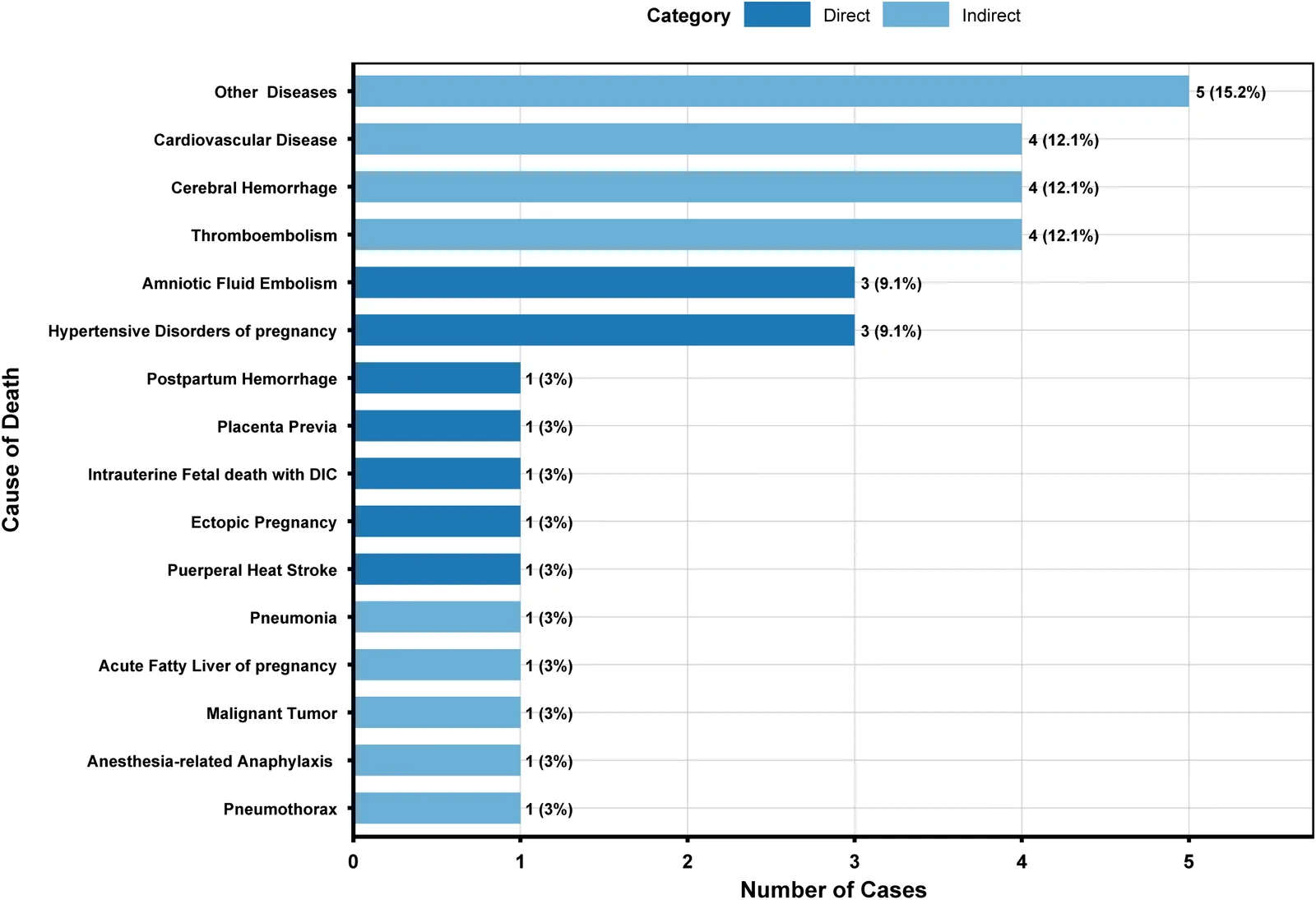
All causes of maternal deaths in Taiyuan (2016–2024) (ranked by number of cases). The number and percentage of maternal deaths by cause during the study period, classified into direct and indirect obstetric causes.

**Fig 9:**
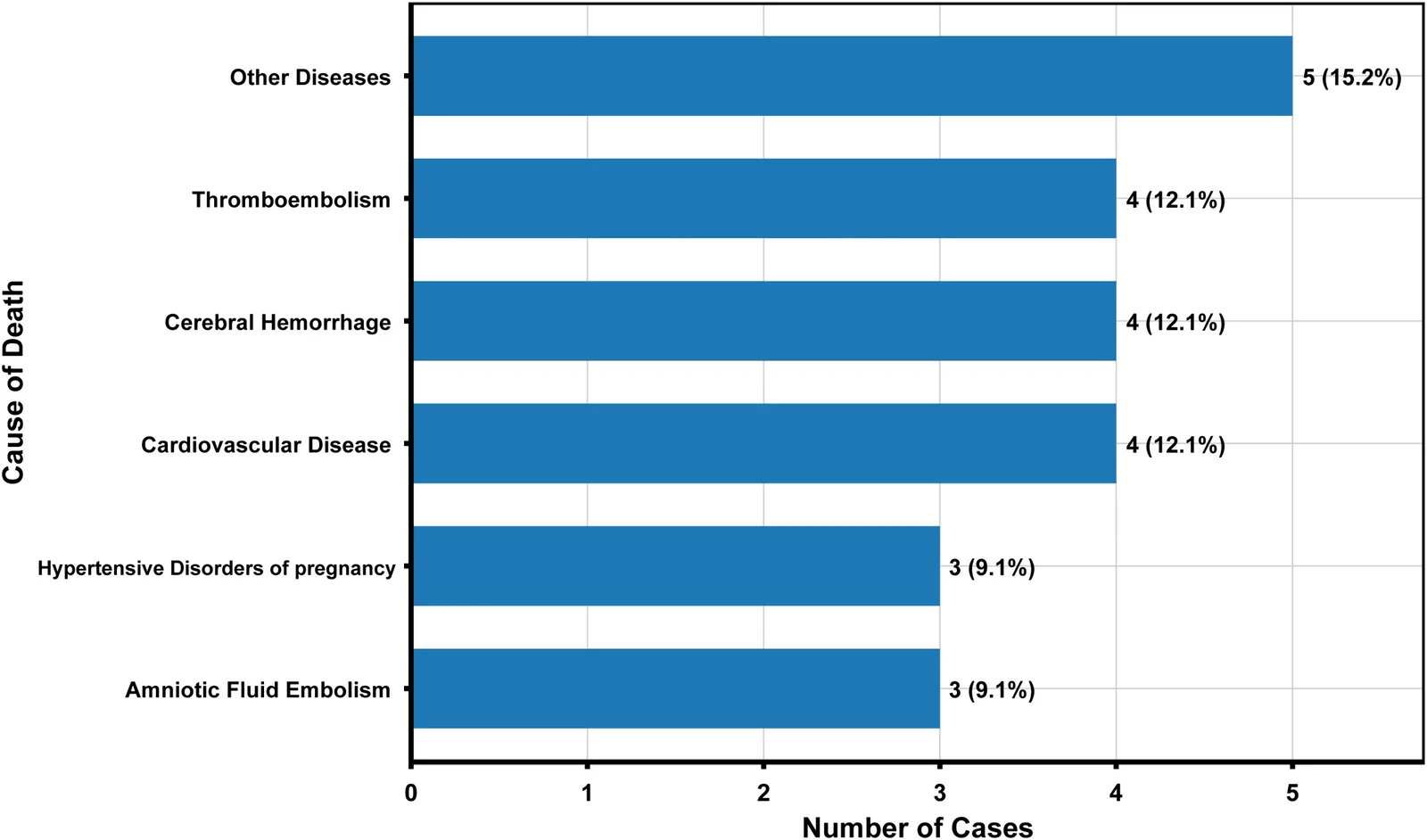
Top six causes of maternal deaths in Taiyuan (2016–2024). The number and proportion of maternal deaths by the six leading causes during the study period.

## Discussion

This study characterizes maternal health indicators in a middle-income Chinese city from 2016 to 2024, a period spanning the implementation of the Two-child and Three-child policies, the COVID-19 pandemic, and sustained fertility decline. The number of pregnant and postpartum women rose modestly before falling steadily after 2020. The overall MMR remained low throughout the study period, with a significant decline observed in urban areas but not in rural settings. Maternal deaths were predominantly attributed to indirect obstetric causes and were concentrated among women aged 30–39 years. These findings suggest a local epidemiological shift away from fatal direct obstetric emergencies toward deaths driven by pre-existing and pregnancy-related comorbidities, alongside persistent urban–rural inequities. A low population-level MMR therefore does not mean that maternal health inequities have been resolved or that preventable deaths have been eliminated.

The decline in the number of pregnant and postpartum women observed in this study parallels national and global fertility trends. Delayed childbearing and a rising prevalence of pregnancies at AMA are well-documented demographic shifts worldwide [15,16]. China's total fertility rate has remained well below replacement level since the mid-1990s and has recently fallen to around one birth per woman [17]. A temporary fertility peak in 2017 likely reflected accumulated reproductive demand released by the universal two-child policy. After 2020, however, the number of pregnant and postpartum women declined steadily. This decline likely reflects a shrinking reproductive-age population, delayed marriage and childbearing, and the COVID-19 pandemic. Rising childcare costs, women’s increasing pursuit of education and careers, and inadequate fertility-support policies may also have contributed, although routine surveillance data cannot separate their effects. These demographic shifts may partly explain why maternal deaths in our study were concentrated among women aged 30–39 years. Stratified analysis showed that women aged 30–34 and 35–39 years accounted for 33.3% and 30.3% of maternal deaths, respectively, with the latter group defined as AMA. This age distribution is consistent with the national trend toward later childbearing and a rising prevalence of AMA after implementation of the universal Two-child and Three-child policies [18].

The trend toward delayed childbearing has been accompanied by a change in the causes of maternal death. Cardiovascular disease and venous thromboembolism together accounted for 66.7% of indirect maternal deaths, and indirect causes overall accounted for nearly two-thirds of total maternal mortality. Heart disease (HD) is one of the leading indirect causes of maternal deaths worldwide, both in high- and low- and middle-income countries [19].AMA substantially increases the risk of preeclampsia and gestational diabetes mellitus and is associated with a higher prevalence of pre-existing chronic conditions [16,20,21], which may partly explain the predominance of medical comorbidities in the region’s mortality profile. This pattern contrasts sharply with that in most low-and middle-income countries, where direct obstetric emergencies such as postpartum hemorrhage remain leading causes of maternal death [22]. This contrast may indicate a structural shift in maternal health challenges in middle-income cities in China. The low burden of direct obstetric mortality in our data likely reflects strong local capacity to manage acute complications, including facility-based delivery, standardized clinical protocols, and established emergency rescue systems [23]. As deaths from acute obstetric events have declined, chronic illness and multimorbidity have become the main threats to maternal safety in urban China. Risks are compounded when AMA coexists with pre-pregnancy chronic disease, obesity, and other metabolic and obstetric risk factors [24]. Maternal health services in this setting should therefore address both acute obstetric risk and the growing burden of chronic comorbidity [25].

Our study also identified substantial urban–rural inequities in maternal health. Although the urban MMR declined substantially, rural MMR showed no statistically significant improvement. This pattern is consistent with the structural imbalance in maternal and child health resources between urban and rural areas in China [9]. Rural regions in central and western provinces, including Shanxi, have long faced weak economic foundations, an understaffed community maternal and child health workforce, and inconsistent service quality [26–29]. Taiyuan has established a city-county network for critically ill pregnant women and a unified urban-rural referral system [30]. 83.9% of maternal deaths occurred in provincial or municipal hospitals, a pattern that may reflect both the concentration of severe cases in tertiary hospitals and delays before referral. Some deaths also occurred outside hospitals or during transfer, indicating gaps in community-level screening and timely identification of high-risk pregnancies, as well as shortages in the community health workforce. Reducing rural maternal mortality will require stronger tertiary emergency care and, equally important, better early screening, early warning, and initial case management at the community level.

The urban—rural gap observed in Taiyuan mirrors patterns of maternal health inequity seen worldwide. Globally, maternal mortality has declined, but wide regional disparities persist, with low- and middle-income countries bearing the greatest burden [31,32]. More than 95% of maternal deaths occur in resource-limited settings, and most are preventable through continuous care across the preconception, antenatal, intrapartum, and postpartum periods [33–36]. By contrast, Taiyuan’s MMR remained below the national average throughout the study period, and even its peak values were lower than the average in high-income countries [37]. This favorable outcome coincides with the local implementation of the FSMNS, first introduced in Taiyuan in 2018 and later scaled up across the province; however, their causal contribution remains to be evaluated.

Since 2025, several secondary and tertiary hospitals in Taiyuan **have been running** standardized early-pregnancy clinics. These clinics provide free **provide free services including early pregnancy confirmation**, risk assessment, nutritional and psychological counseling, and management of abnormal pregnancies. **Given the shift in the causes of maternal death**, future maternal safety strategies in Taiyuan should move beyond emergency-focused obstetric care and include systematic management of comorbidity. Cardiovascular and thromboembolic risk assessment could be integrated into routine preconception care, particularly for women of advanced maternal age, women with obesity, and those with a family history of chronic disease.

Community-based early screening, risk stratification, and referral pathways also need strengthening. Pregnant women of AMA with multimorbidity would benefit from coordinated multidisciplinary care. Standardized clinical protocols, regular quality audits, and optimized referral pathways could reduce preventable maternal deaths and narrow urban-rural health disparities. These findings can guide improvements in maternal health management for similar middle-income cities in China.

### Limitations

This study has some limitations. The number of maternal deaths over the nine-year period was small. As a retrospective analysis based on routine maternal and child health reports, temporal variability in the accuracy and consistency of cause-of-death classification may exist. In addition, the reports lacked sufficient detail to specify the conditions grouped under “other diseases”.We were unable to adjust for key confounders, including socioeconomic status, parity, and specialist care access. Furthermore, overlapping policy reforms, the COVID-19 pandemic, and falling fertility rates precluded clear differentiation of their independent effects; thus, causal inferences cannot be drawn.

## Conclusion

Taiyuan has maintained a low maternal mortality ratio, but indirect obstetric causes now predominate, with deaths concentrated among women aged 30–39 years. Prevention efforts should therefore prioritize this age group. This will require earlier detection and coordinated management of medical and surgical comorbidities, better care for women of AMA, and a shift from obstetric-centered care toward care across the life course. Continued surveillance, targeted resource allocation, and integrated care pathways will be needed to reduce preventable deaths. Taiyuan’s experience offers a useful example for other middle-income urban settings.
