# Supplementary material for: Trends and causes of maternal mortality in Taiyuan, China, 2016–2024: A population-based observational study
Source: PLoS One. 2026 Nov 13;21(11):e0362983. doi: 10.1371/journal.pone.0362983 (PMC13766269; doi:10.1371/journal.pone.0362983)
Supplement: Supplementary file 1 [file pone.0362983.s001.docx]

Reviewer Report
Manuscript Number: PONE-D-26-09796
Title: Trends and Causes of Maternal Mortality in Taiyuan, China, 2016–2024: A Population-Based Observational Study

**Recommendation: Major Revision.**

The dataset is a valuable, apparently carefully maintained, nine-year city-level maternal mortality series that adds real information to the sparse literature on sub-provincial MMR trends in China, and I would be glad to see a revised version. However, the manuscript has several methodological, statistical, and interpretive weaknesses that preclude publication in its current form. In particular, trend tests on rare-event count ratios require appropriate modeling; 95% confidence intervals are entirely absent; causal inferences regarding healthcare policies and clinical quality exceed the observational study design; and ethics and data-sharing statements show critical contradictions. Substantial revision across all sections is necessary.

**Major Revisions**

1. Statistical Modeling and Missing Confidence Intervals

a. Rare-event modeling: The authors apply the Cochran–Armitage trend test to annual maternal mortality ratios (MMRs). Because MMR is a rate based on low Poisson-distributed count data with substantially varying annual live-birth denominators, the Cochran–Armitage test is not appropriate. The authors should implement generalized linear models suitable for rare-event rate data (such as Poisson or negative binomial regression incorporating log (live births) as an offset) to evaluate temporal trends rigorously.

b. Absence of uncertainty estimates: Every reported point estimate including overall, annual, urban/rural, and cause-specific MMRs must be presented alongside exact 95% confidence intervals (e.g., via Poisson exact or score methods). Given that the total dataset consists of only 33 deaths, point estimates such as 0.00 per 100,000 in 2022 and 2024 do not imply zero underlying risk, but rather reflect Poisson variability and limited statistical power

2. Denominator and Risk Interpretation for Maternal Age

In Section 3 of the Results and the Discussion, the authors assert that women aged 30–39 years face the greatest vulnerability because they represent 63.6% (21/33) of maternal deaths. Proportions of total deaths (constituent ratios) reflect underlying maternal age distribution in the birthing population and do not measure individual risk. The authors must either obtain age-stratified live birth counts to compute true age-specific MMRs with 95% CIs, or explicitly reframe this section as a descriptive distribution of deaths without asserting elevated age-specific risk.

3. Over-interpretation and Causal Claims

The Discussion frequently attributes temporal declines and hospital-based death patterns to specific policy initiatives (e.g., the Five Strategies for Maternal and Newborn Safety) and tertiary care quality.

Tone down all causal assertions

The concentration of deaths in tertiary hospitals (83.9%) likely reflects referral bias and the transfer of critically ill patients rather than hospital-level treatment efficacy. The authors must present this alternative explanation and qualify conclusions accordingly.

4. Diagnostic and Cause-of-Death Classification

In Table 4 and Figure 9, "Stillbirth" is listed as a direct maternal cause of death (1 case, 3.0%). Stillbirth is a fetal outcome, not a maternal etiology of death; this classification must be reviewed and re-coded according to the WHO Application of ICD-10 to Deaths during Pregnancy, Childbirth and the Puerperium (ICD-MM)

Other non-standard or broad categories (e.g., "Puerperal Heat Stroke", "Other Medical Diseases", and "Anesthesia Drug Allergy") require explicit clinical definitions and formal ICD-MM coding. The manuscript should specify whether cases underwent formal confidential maternal mortality committee audits to adjudicate primary cause of death.

5. . Ethics Approval and Data Availability Inconsistencies

In the submission metadata, the authors declare "N/A" for ethics because the data were sourced from "publicly available statistical reports". However, the Methods section explicitly states that records were retrieved from the Taiyuan Maternal Monitoring Information System and the Maternal and Child Health Information Network Direct Reporting System, containing case-level clinical summaries.

The authors must provide the formal name of the Institutional Review Board (IRB) or Ethics Committee, the protocol approval/waiver number, and documentation of informed consent or consent waiver/exemption.

The Data Availability Statement must clarify access terms; individual health surveillance records with sensitive clinical data cannot be labeled "fully available without restriction" without violating participant privacy policies.

6. Urban–Rural Comparisons and Subgroup Consistency

a. The authors report an urban MMR of 7.58 vs. a rural MMR of 9.57 per 100,000, noting no overall significant difference, yet report a significant difference for 2016–2018 in Figure 3.

b. With only 4 rural deaths across the entire 9-year study window, rural subgroup analyses are prone to extreme small-sample instability. The authors should report exact test statistics, $2\times2$contingency tables, and exact rate ratios with 95%

**Minor Revisions**

1. Abstract: Remove generic background phrasing (e.g., "Women play an indispensable role in both family and society") and begin directly with the epidemiological problem and research objective.

2. Terminology: Replace the inaccurate term "maternal mortality rate" with maternal mortality ratio (MMR) throughout the entire text, tables, and figure axes, as the denominator is live births. Replace vague terms like "maternal patients" with precise clinical descriptors (e.g., pregnant women or live births).

3. Table and Figure Discrepancies:

Reconcile figure numbering between the text (Figures 1–9) and uploaded files (which include up to Figure 10).

In Table 3, the sum of location-specified deaths appears to be 31 rather than 33. Clearly report the number of missing/unspecified cases and ensure all percentages use a consistent, documented denominator.

4. Funding and Declarations: Reconcile grant numbers and funder names between the manuscript title page and the submission form metadata.

5. Language and Copyediting: Correct typographical errors and unstandardized abbreviations (e.g., "National Key Clinica Specialty", "Programmers", and irregular spacing around punctuation) throughout the manuscript. Ensure that all reference citations adhere strictly to journal formatting guidelines
