# Supplementary material for: Trends and causes of maternal mortality in Taiyuan, China, 2016–2024: A population-based observational study
Source: PLoS One. 2026 Nov 13;21(11):e0362983. doi: 10.1371/journal.pone.0362983 (PMC13766269; doi:10.1371/journal.pone.0362983)
Supplement: Peer review history

# Peer review: Decision Letter 0

**DOI:** 10.1371/journal.pone.0362983.r001

**Authors:** Adewale Olufemi Ashimi

27 Aug 2026

Dear Dr. Yang,

Thank you for submitting your manuscript to PLOS One. After careful consideration, we feel that it has merit but does not fully meet PLOS One’s publication criteria as it currently stands. Therefore, we invite you to submit a revised version of the manuscript that addresses the points raised during the review process.

Please submit your revised manuscript by Oct 11 2026 11:59PM. If you will need more time than this to complete your revisions, please reply to this message or contact the journal office at plosone@plos.org. When you're ready to submit your revision, log on to https://www.editorialmanager.com/pone/ and select the 'Submissions Needing Revision' folder to locate your manuscript file.

Please include the following items when submitting your revised manuscript:

- A letter that responds to each point raised by the academic editor and reviewer(s). You should upload this letter as a separate file labeled 'Response to Reviewers'.
- A marked-up copy of your manuscript that highlights changes made to the original version. You should upload this as a separate file labeled 'Revised Manuscript with Track Changes'.
- An unmarked version of your revised paper without tracked changes. You should upload this as a separate file labeled 'Manuscript'.

If you would like to make changes to your financial disclosure, please include your updated statement in your cover letter. Guidelines for resubmitting your figure files are available below the reviewer comments at the end of this letter.

If applicable, we recommend that you deposit your laboratory protocols in protocols.io to enhance the reproducibility of your results. Protocols.io assigns your protocol its own identifier (DOI) so that it can be cited independently in the future. For instructions see: https://journals.plos.org/plosone/s/submission-guidelines#loc-laboratory-protocols. Additionally, PLOS One offers an option for publishing peer-reviewed Lab Protocol articles, which describe protocols hosted on protocols.io. Read more information on sharing protocols at https://plos.org/protocols?utm_medium=editorial-email&utm_source=authorletters&utm_campaign=protocols.

As the corresponding author, your ORCID iD is verified in the submission system and will appear in the published article. PLOS supports the use of ORCID, and we encourage all coauthors to register for an ORCID iD and use it as well. Please encourage your coauthors to verify their ORCID iD within the submission system before final acceptance, as unverified ORCID iDs will not appear in the published article. *Only*  the individual author can complete the verification step; PLOS staff *cannot*  verify ORCID iDs on behalf of authors.

We look forward to receiving your revised manuscript.

Kind regards,

Adewale Olufemi Ashimi, MBChB, MPH, PhD, FWACS

Academic Editor

PLOS One

Journal requirements:

When submitting your revision, we need you to address these additional requirements.

1. Please ensure that your manuscript meets PLOS ONE's style requirements, including those for file naming. The PLOS ONE style templates can be found at

https://journals.plos.org/plosone/s/file?id=wjVg/PLOSOne_formatting_sample_main_body.pdf and

https://journals.plos.org/plosone/s/file?id=ba62/PLOSOne_formatting_sample_title_authors_affiliations.pdf

2. Thank you for stating the following financial disclosure:

“This work was supported by the Science and Technology Innovation Base ProjectConstruction Task ofShanxi Province (Grant No.2024-ZZ-006), the National Key Clinica Specialty Construction Project of Shanxi Province (Grant No.YDZJSX2022B010)”

Please state what role the funders took in the study.  If the funders had no role, please state: "The funders had no role in study design, data collection and analysis, decision to publish, or preparation of the manuscript."

If this statement is not correct you must amend it as needed.

Please include this amended Role of Funder statement in your cover letter; we will change the online submission form on your behalf.

3. Thank you for stating the following in your Competing Interests section:

“No.”

Please complete your Competing Interests on the online submission form to state any Competing Interests. If you have no competing interests, please state "The authors have declared that no competing interests exist.", as detailed online in our guide for authors at http://journals.plos.org/plosone/s/submit-now

This information should be included in your cover letter; we will change the online submission form on your behalf.

If the reviewer comments include a recommendation to cite specific previously published works, please review and evaluate these publications to determine whether they are relevant and should be cited. There is no requirement to cite these works unless the editor has indicated otherwise.

[Note: HTML markup is below. Please do not edit.]

Reviewers' comments:

Reviewer's Responses to Questions

**Comments to the Author**

1. Is the manuscript technically sound, and do the data support the conclusions?

Reviewer #1: Yes

Reviewer #2: Partly

**********

2. Has the statistical analysis been performed appropriately and rigorously?

Reviewer #1: Yes

Reviewer #2: No

**********

3. Have the authors made all data underlying the findings in their manuscript fully available?

The PLOS Data policy

Reviewer #1: Yes

Reviewer #2: Yes

**********

4. Is the manuscript presented in an intelligible fashion and written in standard English?

Reviewer #1: Yes

Reviewer #2: No

**********

Reviewer #1: This is a good and timely manuscript written to help us understand the trends and causes of maternal mortality in China. The manuscript has also been nicely written, with only a few minor comments below, that could help improve the manuscript.

1. The authors have given the limitations of the study but did not give the strengths. It is important to mention some of the strengths of the study just before the limitations.

2. What are some of the ethical considerations you followed for this study? Did you receive any ethical approval from a Research Ethics Committee?

Reviewer #2: This manuscript analyzes population-based maternal mortality surveillance data from Taiyuan, China, spanning 2016 through 2024 (33 maternal deaths among 424,392 live births). The study addresses an essential topic in reproductive epidemiology and maternal healthcare delivery, offering rare city-level surveillance insights. The finding that indirect obstetric causes account for two-thirds of maternal deaths is clinically and programmatically relevant. Please refer to the attachment.

**********

what does this mean?). If published, this will include your full peer review and any attached files.

If you choose “no”, your identity will remain anonymous but your review may still be made public.

**Do you want your identity to be public for this peer review?**  For information about this choice, including consent withdrawal, please see our Privacy Policy

Reviewer #1: No

Reviewer #2: **Yes:**  Anum Aziz

**********

[NOTE: If reviewer comments were submitted as an attachment file, they will be attached to this email and accessible via the submission site. Please log into your account, locate the manuscript record, and check for the action link "View Attachments". If this link does not appear, there are no attachment files.]

To ensure your figures meet our technical requirements, please review our figure guidelines: https://journals.plos.org/plosone/s/figures

You may also use PLOS’s free figure tool, NAAS, to help you prepare publication quality figures: https://journals.plos.org/plosone/s/figures#loc-tools-for-figure-preparation.

NAAS will assess whether your figures meet our technical requirements by comparing each figure against our figure specifications.

Attachment Submitted filename: Reviewer Report- PLOS ONE PONE-D-26-09796.docx

# Author comment: Author response to Decision Letter 1

**DOI:** 10.1371/journal.pone.0362983.r002

8 Oct 2026

Dear Editors and Reviewers:

Thank you for the comments about our manuscript entitled " Trends and Causes of Maternal Mortality in Taiyuan, China, 2016–2024: A Population-Based Observational Study " (Manuscript ID: PONE-D-26-09796). The comments are constructive to improve our paper. We have discussed them and tried to address all the points according to the comments. Revised sentences are marked in blue in the manuscript. The responses to the reviewer's comments are as below.

Reviewer: 1

Comments to the Author

This is a good and timely manuscript written to help us understand the trends and causes of maternal mortality in China. The manuscript has also been nicely written, with only a few minor comments below, that could help improve the manuscript.

Comment 1：The authors have given the limitations of the study but did not give the strengths. It is important to mention some of the strengths of the study just before the limitations.

Response：Thank you very much for your review and evaluation of the manuscript.

We have added a paragraph describing the strengths of this study at the beginning of the Discussion section.The newly added text emphasizes our strengths: nine consecutive years of city-level maternal mortality surveillance data and stratified urban—rural disparity analysis, which provides region-specific evidence for maternal health policy.

Comment 2：What are some of the ethical considerations you followed for this study? Did you receive any ethical approval from a Research Ethics Committee?

Response：Ethical approval for this retrospective observational study was obtained from the Research Ethics Committee of The First Hospital of Shanxi Medical University. The ethical approval number is KYLL-2026-439. All data used in this study were de-identified aggregated routine surveillance data, with no access to individual identifiable personal information. We have supplemented this information in the Ethics statement section of the manuscript.

Reviewer: 2

Comments to the Author

The dataset is a valuable, apparently carefully maintained, nine-year city-level maternal mortality series that adds real information to the sparse literature on sub-provincial MMR trends in China, and I would be glad to see a revised version. However, the manuscript has several methodological, statistical, and interpretive weaknesses that preclude publication in its current form. In particular, trend tests on rare-event count ratios require appropriate modeling; 95% confidence intervals are entirely absent; causal inferences regarding healthcare policies and clinical quality exceed the observational study design; and ethics and data-sharing statements show critical contradictions. Substantial revision across all sections is necessary

Response: Thank you very much for your positive assessment of our city-level maternal mortality dataset and your comprehensive critical comments. We fully acknowledge these important shortcomings in the original manuscript. We have substantially revised the Methods, Results and Discussion sections accordingly, as detailed in the following point-by-point replies.

Comment 1：Statistical Modeling and Missing Confidence Intervals.

Response: We thank the reviewer for this critical methodological suggestion.

a.Rare-event modeling: We have replaced the Cochran–Armitage trend test with Poisson regression models incorporating the natural logarithm of annual live births as an offset term. Overdispersion was assessed using the Pearson chi-square statistic divided by its degrees of freedom; no significant overdispersion was detected (X²/df = 0.627), confirming the appropriateness of the Poisson model.

The annual percent change (APC) with its 95% confidence interval is now reported instead of the Cochran-Armitage test statistic. The revised Methods section (page 6-7) and Results section (page 7-15) have been updated accordingly.

b.Absence of uncertainty estimates: We have now computed exact 95% confidence intervals for all reported MMRs using the Poisson exact method. All tables (Tables 1-5) now include a “95% CI” column for each MMR estimate, and all point estimates in the main text are now presented with their corresponding 95% CIs (e.g., “overall MMR: 7.78 [95% CI: 5.35 to 10.92] per 100,000 live births”).

For years with zero maternal deaths (2022 and 2024), we now report the MMR as “0.00 (95% CI: 0.00 to 8.92)”-explicitly acknowledging that zero events do not equate to zero risk.

Summary of Key Results

Category APC 95% CI P-value

Overall MMR -29.83% (-41.22%, -16.23%) 0.0001

Urban areas -30.89% (-42.98%, -16.23%) 0.0002

Rural areas -22.70% (-51.05%, 22.06%) 0.2693

Direct obstetric causes -40.00% (-58.58%, -13.09%) 0.0069

Indirect obstetric causes -25.43% (-39.09%, -8.69%) 0.0045

Overall MMR: 7.78 per 100,000 live births (95% CI: 5.35 to 10.92)

Comment 2：Denominator and Risk Interpretation for Maternal Age

In Section 3 of the Results and the Discussion, the authors assert that women aged 30–39 years face the greatest vulnerability because they represent 63.6% (21/33) of maternal deaths. Proportions of total deaths (constituent ratios) reflect underlying maternal age distribution in the birthing population and do not measure individual risk. The authors must either obtain age-stratified live birth counts to compute true age-specific MMRs with 95% CIs, or explicitly reframe this section as a descriptive distribution of deaths without asserting elevated age-specific risk.

Response:We appreciate this critical methodological feedback. Unfortunately, our dataset does not allow for fine-scale, age-stratified live-birth denominators at the municipal level across the study period. Accordingly, we have fully removed the previous statement suggesting an elevated individual risk among women aged 30–39 years. This section has been revised to provide a purely descriptive presentation of the age distribution among observed maternal deaths in our study.

Revised Results

The 30–34 years age group had the highest number of maternal deaths (11 cases, 33.3%), followed closely by the 35–39 years age group (10 cases, 30.3%). Overall, women aged 30–39 years accounted for 21 of the 33 total maternal deaths, representing 63.6% of all fatalities observed during the study period (Fig 6).

Revised Discussion

These demographic shifts may partly explain the concentration of maternal deaths among women aged 30–39 years in our cohort. Stratified analysis indicated that women aged 30–34 and 35–39 years contributed to 33.3% and 30.3% of all maternal deaths, respectively, with the latter group defined as AMA. This age distribution is consistent with the national trend of delayed childbearing and the increased prevalence of AMA following the implementation of the universal Two-child and Three-child policies in China [18].

Comment 3：Over-interpretation and Causal Claims

The Discussion frequently attributes temporal declines and hospital-based death patterns to specific policy initiatives (e.g., the Five Strategies for Maternal and Newborn Safety) and tertiary care quality.Tone down all causal assertions.The concentration of deaths in tertiary hospitals (83.9%) likely reflects referral bias and the transfer of critically ill patients rather than hospital-level treatment efficacy. The authors must present this alternative explanation and qualify conclusions accordingly.

Response: Thank you for pointing out this common pitfall in observational study interpretation. We have toned down all causal language throughout the Discussion section. We removed wording that directly links MMR changes to policy interventions or hospital treatment effectiveness.

Revised Discussion

Taiyuan has established a city-county network for critically ill pregnant women and a unified urban-rural referral system [30]. 83.9% of maternal deaths occurred in provincial or municipal hospitals, a pattern that may reflect both the concentration of severe cases in tertiary hospitals and delays before referral. Some deaths also occurred outside hospitals or during transfer, indicating gaps in community-level screening and timely identification of high-risk pregnancies, as well as shortages in the community health workforce.

Comment 4：Diagnostic and Cause-of-Death Classification

In Table 4 and Figure 9, "Stillbirth" is listed as a direct maternal cause of death (1 case, 3.0%). Stillbirth is a fetal outcome, not a maternal etiology of death; this classification must be reviewed and re-coded according to the WHO Application of ICD-10 to Deaths during Pregnancy, Childbirth and the Puerperium (ICD-MM)

Other non-standard or broad categories (e.g., "Puerperal Heat Stroke", "Other Medical Diseases", and "Anesthesia Drug Allergy") require explicit clinical definitions and formal ICD-MM coding. The manuscript should specify whether cases underwent formal confidential maternal mortality committee audits to adjudicate primary cause of death.

Response: We thank the reviewer for this careful check on cause-of-death coding.

In accordance with the WHO ICD-MM guidance, we have re-evaluated and re-coded the case previously classified as “Stillbirth.” The maternal death has been reclassified as “Intrauterine fetal death with DIC,” with the underlying maternal cause attributed to DIC secondary to intrauterine fetal death.

Upon verification, we have made the following corrections:

“Puerperal Heat Stroke” was confirmed as “Puerperal Heatstroke”;

“Anesthesia Drug Allergy” has been revised to “Anesthesia-related Anaphylaxis”;

“Idiopathic Fatty Liver” has been revised to “Acute Fatty Liver of Pregnancy.”

Regarding “Other Medical Diseases,” the Taiyuan Maternal and Child Health Annual Report lacked sufficient detail to specify the individual conditions grouped under this broad category. We have therefore explicitly acknowledged this limitation in the Limitations section.

Table 4 and Figure 9 have been updated accordingly.

We have added a clear statement in the Quality Control section: The local maternal mortality review committee conducts reviews every six months; each case undergoes a standardized review, and an individual report is prepared to identify the most likely cause of death, preventable factors, and recommended interventions. Causes of death were coded according to ICD-10.

Comment 5：Ethics Approval and Data Availability Inconsistencies

In the submission metadata, the authors declare "N/A" for ethics because the data were sourced from "publicly available statistical reports". However, the Methods section explicitly states that records were retrieved from the Taiyuan Maternal Monitoring Information System and the Maternal and Child Health Information Network Direct Reporting System, containing case-level clinical summaries.

The authors must provide the formal name of the Institutional Review Board (IRB) or Ethics Committee, the protocol approval/waiver number, and documentation of informed consent or consent waiver/exemption.

The Data Availability Statement must clarify access terms; individual health surveillance records with sensitive clinical data cannot be labeled "fully available without restriction" without violating participant privacy policies.

Response: We sincerely apologize for this inconsistency in our original submission. We have supplemented the ethics information and have revised the Materials and methods section.Ethical approval for this retrospective observational study was obtained from the Research Ethics Committee of The First Hospital of Shanxi Medical University. The ethical approval number is KYLL-2026-439. All data used in this study were de-identified aggregated routine surveillance data, with no access to individual identifiable personal information. We have supplemented this information in the Ethics statement section of the manuscript.

Revised Data Source

Data were extracted from the Taiyuan Maternal and Child Health Annual Statistical Report. Routine, population-based maternal mortality surveillance was implemented in Taiyuan following the Shanxi Provincial Maternal Mortality Monitoring Program and Taiyuan Municipal Implementation Rules for Maternal Mortality Monitoring. Full-time staff at the Taiyuan Maternal Health and Family Planning Service Center performed surveillance data collection, quality control, statistical analysis, and municipal-level maternal mortality reviews; aggregated findings from this surveillance were compiled into the annual report. We analysed live births and maternal deaths reported by all medical institutions across Taiyuan.

The data availability statement is retained because the dataset includes no sensitive clinical records or individually identifiable health surveillance data.

Comment 6：Urban–Rural Comparisons and Subgroup Consistency

a. The authors report an urban MMR of 7.58 vs. a rural MMR of 9.57 per 100,000, noting no overall significant difference, yet report a significant difference for 2016–2018 in Figure 3.

b. With only 4 rural deaths across the entire 9-year study window, rural subgroup analyses are prone to extreme small-sample instability. The authors should report exact test statistics, 2×2contingency tables, and exact rate ratios with 95%

Response: We appreciate this thoughtful comment.

a.Chi-square analyses indicated no statistically significant differences in MMR between urban and rural areas for the period 2016–2024, whereas subgroup analysis revealed a statistically significant urban–rural difference in MMR during 2016–2018 (P < 0.001).

b.We performed Poisson regression analysis with 95% confidence intervals. The overall MMR demonstrated a significant downward trend during the study period (APC = −29.83%, 95% CI: −41.22% to −16.23%, P = 0.0001). Urban areas showed a significant decline (APC = −30.89%, 95% CI: −42.98% to −16.23%, P = 0.0002), while rural areas had no statistically significant trend (APC = −22.70%, 95% CI: −51.05% to 22.06%, P = 0.2693).

Minor Revisions

Comment 1：Abstract

Remove generic background phrasing (e.g., "Women play an indispensable role in both family and society") and begin directly with the epidemiological problem and research objective.

Response: Thanks for your comments.The generic introductory sentence has been deleted from Abstract.

Comment 2：Terminology

Replace the inaccurate term "maternal mortality rate" with maternal mortality ratio (MMR) throughout the entire text, tables, and figure axes, as the denominator is live births. Replace vague terms like "maternal patients" with precise clinical descriptors (e.g., pregnant women or live births).

Response: We greatly appreciate your insightful suggestions.We have performed full text search-and-replace. The term “maternal mortality rate” is completely replaced by maternal mortality ratio (MMR) across main text, all table captions, table content and all figure axes. Vague term “maternal patients” is replaced with live births.

Comment 3. Table and Figure Discrepancies

a.Reconcile figure numbering between the text (Figures 1–9) and uploaded files (which include up to Figure 10).

b.In Table 3, the sum of location-specified deaths appears to be 31 rather than 33. Clearly report the number of missing/unspecified cases and ensure all percentages use a consistent, documented denominator.

Response:

a.We have harmonized figure numbering: removed redundant Figure 10, and aligned all in-text figure citations exactly with uploaded figure files (Figures 1-9).For Table 3: b.We verified that the sum of location-specified deaths in Table 3 was indeed 33.

4. Funding and Declarations

Reconcile grant numbers and funder names between the manuscript title page and the submission form metadata.

Response: We have reconcile grant numbers and funding agency names in manuscript title page and submission metadata. As requested by the editorial office, we add the statement: The funders had no role in study design, data collection and analysis, decision to publish, or preparation of the manuscript in Funding section.

5.Language and Copyediting

Correct typographical errors and unstandardized abbreviations (e.g., "National Key Clinica Specialty", "Programmers", and irregular spacing around punctuation) throughout the manuscript. Ensure that all reference citations adhere strictly to journal formatting guidelines.

Response: We greatly appreciate your valuable feedback.We have carefully proof-read the full manuscript. Typographical mistakes including “National Key Clinica Specialty” are corrected. We fixed abnormal punctuation spacing, standardized all abbreviations. All reference list entries have been reformatted to comply with PLOS ONE citation format requirements.

Editorial office requirements

1.Funding role statement: As requested, cover letter and manuscript acknowledgements include: “The funders had no role in study design, data collection and analysis, decision to publish, or preparation of the manuscript.”

2.Conflict of interest: We have updated the conflict-of-interest statement as: The authors have declared that no competing interests exist. This statement is presented both in cover letter and revised manuscript.

We tried our best to improve the manuscript and made some changes marked in revised paper which will not influence the content and framework of the paper. We appreciate for Editors/Reviewers’ comments earnestly, and hope the correction will meet with approval. Once again, thank you very much for your comments and suggestions.

Your sincerely,

Hailan Yang

Attachment Submitted filename: Response to Reviewers-PLOS ONE PONE-D-26-09796.doc

# Peer review: Decision Letter 1

**DOI:** 10.1371/journal.pone.0362983.r003

**Authors:** Adewale Olufemi Ashimi

3 Nov 2026

<p>Trends and Causes of Maternal Mortality in Taiyuan, China, 2016–2024: A Population-Based Observational Study

PONE-D-26-09796R1

Dear Dr. Yang,

We’re pleased to inform you that your manuscript has been judged scientifically suitable for publication and will be formally accepted for publication once it meets all outstanding technical requirements.

Within one week, you’ll receive an e-mail detailing the required amendments. When these have been addressed, you’ll receive a formal acceptance letter and your manuscript will be scheduled for publication.

An invoice will be generated when your article is formally accepted. Please note, if your institution has a publishing partnership with PLOS and your article meets the relevant criteria, all or part of your publication costs will be covered. Please make sure your user information is up-to-date by logging into Editorial Manager at Editorial Manager® and clicking the ‘Update My Information' link at the top of the page. For questions related to billing, please contact billing support.

If your institution or institutions have a press office, please notify them about your upcoming paper to help maximize its impact. If they’ll be preparing press materials, please inform our press team as soon as possible -- no later than 48 hours after receiving the formal acceptance. Your manuscript will remain under strict press embargo until 2 pm Eastern Time on the date of publication. For more information, please contact onepress@plos.org.

Kind regards,

Adewale Olufemi Ashimi, MBChB, MPH, PhD, FWACS

Academic Editor

PLOS One

Additional Editor Comments (optional):

Reviewers' comments:

Reviewer's Responses to Questions

**Comments to the Author**

Reviewer #1: All comments have been addressed

Reviewer #2: All comments have been addressed

**********

2. Is the manuscript technically sound, and do the data support the conclusions?

Reviewer #1: Yes

Reviewer #2: Yes

**********

3. Has the statistical analysis been performed appropriately and rigorously?

Reviewer #1: Yes

Reviewer #2: Yes

**********

4. Have the authors made all data underlying the findings in their manuscript fully available?

The PLOS Data policy

Reviewer #1: Yes

Reviewer #2: Yes

**********

5. Is the manuscript presented in an intelligible fashion and written in standard English?

Reviewer #1: Yes

Reviewer #2: Yes

**********

Reviewer #1: The authors have done a great job addressing all the comments they were given. I have no further comments.

Reviewer #2: Dear Author,

Thank you for revising the script and now it sounds more scientific.

All queries have been asnwered satisfactorily.

Thanks.

**********

what does this mean?). If published, this will include your full peer review and any attached files.

If you choose “no”, your identity will remain anonymous but your review may still be made public.

**Do you want your identity to be public for this peer review?**  For information about this choice, including consent withdrawal, please see our Privacy Policy

Reviewer #1: No

Reviewer #2: No

**********

# Editor report: Acceptance letter

**DOI:** 10.1371/journal.pone.0362983.r004

**Authors:** Adewale Olufemi Ashimi

PONE-D-26-09796R1

PLOS One

Dear Dr. Yang,

I'm pleased to inform you that your manuscript has been deemed suitable for publication in PLOS One. Congratulations! Your manuscript is now being handed over to our production team.

At this stage, our production department will prepare your paper for publication. This includes ensuring the following:

* All references, tables, and figures are properly cited

* All relevant supporting information is included in the manuscript submission,

* There are no issues that prevent the paper from being properly typeset

You will receive further instructions from the production team, including instructions on how to review your proof when it is ready. Please keep in mind that we are working through a large volume of accepted articles, so please give us a few days to review your paper and let you know the next and final steps.

Lastly, if your institution or institutions have a press office, please let them know about your upcoming paper now to help maximize its impact. If they'll be preparing press materials, please inform our press team within the next 48 hours. Your manuscript will remain under strict press embargo until 2 pm Eastern Time on the date of publication. For more information, please contact onepress@plos.org.

You will receive an invoice from PLOS for your publication fee after your manuscript has reached the completed accept phase. If you receive an email requesting payment before acceptance or for any other service, this may be a phishing scheme. Learn how to identify phishing emails and protect your accounts at https://explore.plos.org/phishing.

If we can help with anything else, please email us at customercare@plos.org.

Thank you for submitting your work to PLOS One and supporting open access.

Kind regards,

PLOS One Editorial Office Staff

on behalf of

Dr. Adewale Olufemi Ashimi

Academic Editor

PLOS One
